# Gene Identification in Inflammatory Bowel Disease via a Machine Learning Approach

**DOI:** 10.3390/medicina59071218

**Published:** 2023-06-28

**Authors:** Gerardo Alfonso Perez, Raquel Castillo

**Affiliations:** Biocomp Group, Institute of Advanced Materials (INAM), Universitat Jaume I, 12071 Castello, Spain

**Keywords:** inflammatory bowel disease, Crohn’s disease, ulcerative colitis

## Abstract

Inflammatory bowel disease (IBD) is an illness with increasing prevalence, particularly in emerging countries, which can have a substantial impact on the quality of life of the patient. The illness is rather heterogeneous with different evolution among patients. A machine learning approach is followed in this paper to identify potential genes that are related to IBD. This is done by following a Monte Carlo simulation approach. In total, 23 different machine learning techniques were tested (in addition to a base level obtained using artificial neural networks). The best model identified 74 genes selected by the algorithm as being potentially involved in IBD. IBD seems to be a polygenic illness, in which environmental factors might play an important role. Following a machine learning approach, it was possible to obtain a classification accuracy of 84.2% differentiating between patients with IBD and control cases in a large cohort of 2490 total cases. The sensitivity and specificity of the model were 82.6% and 84.4%, respectively. It was also possible to distinguish between the two main types of IBD: (1) Crohn’s disease and (2) ulcerative colitis.

## 1. Introduction

In this paper, the genetic expression signature of inflammatory bowel disease is analyzed using machine learning techniques. Inflammatory bowel disease (IBD) is a chronic [1] inflammatory disease, whose cause remains unclear. Patients can show an array of different symptoms. According to the Mayo Clinic, some of the most common symptoms associated with inflammatory bowel disease include pain, diarrhea, fatigue, cramps, blood present in stools and weight loss. Extraintestinal symptoms appear in approximately 24% of patients [2]. Patients can also have very different evolution and responses to treatments.

Another interesting characteristic of this illness, so far without a good explanation, is that it tends to have a higher incidence and prevalence in urban areas [3] compared to rural areas, perhaps suggesting a link to lifestyles. The incidence of IBD has been increasing [4]. Inflammatory bowel disease is becoming an increasingly important health problem [5]. Developing and newly industrialized countries are seeing a particularly rapid increase in the incidence of the illness [6]. The reasons behind this increase remain unclear. It might be related to changes in dietary habits or exposure to pollutants, but there are currently, to the best of our knowledge, no definitive data to prove it. It is also likely that the illness is being detected earlier in those countries as their healthcare infrastructure develops. Nevertheless, environmental factors appear to play a role in the illness. IBD increases the chances of developing other illnesses, such as colorectal cancer [7] and osteoporosis [8]. More than 7% of patients with IBD develop osteoporosis [8]. Additionally, IBD can have a very significant impact on the quality of life of the patient and can make normal activities, such as working, challenging in some severe cases.

**Figure 1:**
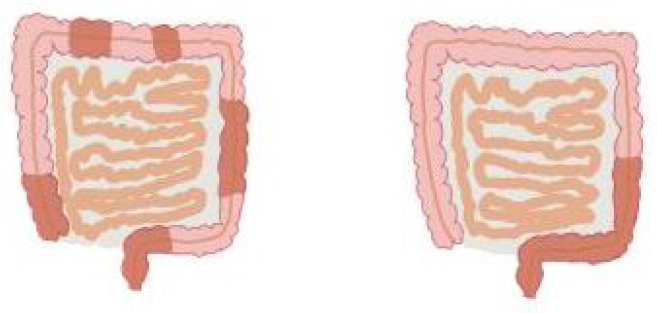
Visual representation of Crohn’s disease (**left**) and ulcerative colitis (**right**). It can be seen some of the usual areas involved in UC and CD. It should be noted that there is substantial variation among patients.

One of the main theories of the cause of IBD is that it is an abnormal immune response in genetically predisposed individuals, triggered by some external factor such as a virus or bacteria [9,10]. Cytokines appear to play an important role in IBD [11]. Lifestyle factors, such as stress, smoking and diet [12], have also been identified in the literature as having a role in the illness [13]. The illness results in a defective regulation of the mucosa [14]. Tamboli et al. [15] specifically mentioned intestinal bacteria as a major factor in the initial stages of the disease. Chang [16] concluded that the two causative agents are (1) abnormal immune response in the gastrointestinal mucosa and (2) alterations in the gut microbiome [17]. The two major forms of IBD are ulcerative colitis (UC) and Crohn’s disease (CD) [18]. A visual representation of UC and CD is shown in Figure 1.

**Figure 2:**
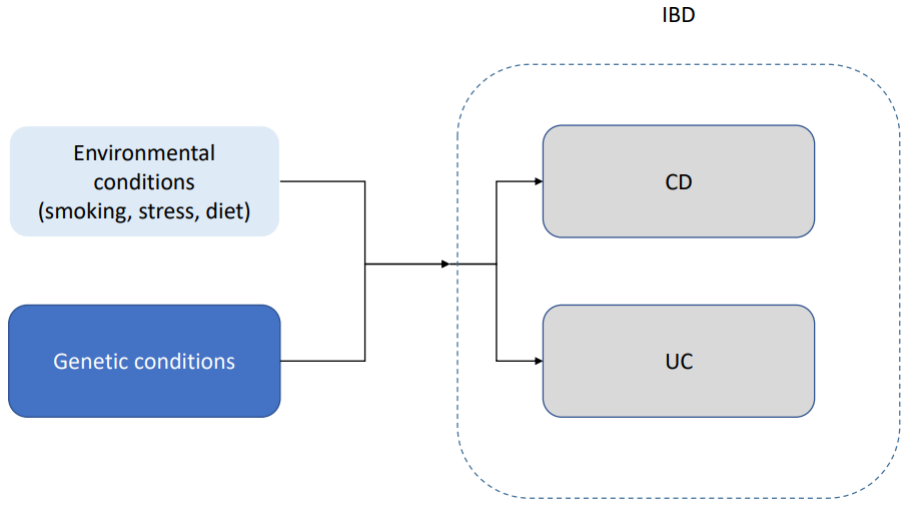
Schematic representation of the interaction between genetic predisposition and environmental factors in ulcerative colitis (UC) and Crohn’s disease (CD). IBD, in both of its main forms, is likely caused by a combination of underlying genetic conditions and environmental conditions.

IBD appears to have a genetic component. Loddo and Romano [19] mentioned that approximately 15% of the patients with Crohn’s disease have a family member with the same condition. They also mentioned a 50% concordance in monozygotic twins. Bernard and Ramnik [20] concluded that genes help regulate the complex interaction between microbial and environmental factors. Another indications of a genetic component in the disease is that some ethnic groups, such as Ashkenazim, have higher incidence and prevalence [21]. Some authors, such as McGovern et al. [22], highlighted the issue that a large amount of the existing literature focuses on individuals of European ancestry. This is especially important in an illness such as IBD, in which ethnicity seems to play an important role not only in terms of prevalence but also in terms of early onset, reaction to the treatment and severity of the illness. A schematic representation of the interaction between genetic predisposition and environmental factors is shown in Figure 2. The underlying mechanics of this interaction between genetic predisposition and environmental factors remain not well understood.

There have been many developments in the genetics of IBD, but despite the identification of some genes, the underlying process remains not well understood. The evidence points to a process in which multiple genes are involved (polygenic) [23,24]. Cho and Abraham [25] cited the well-known Nod2 (CARD15) polymorphism association with Crohn’s disease. This gene is located in chromosome 16 and has been mentioned by multiple authors [26]. Katuka et al. [27] mentioned that in Japan, the NUDT15 polymorphism is routinely tested before administering thiopurine to inflammatory bowel disease patients. Mathew and Lewis [28] studied genes in chromosome 5q31n 6p21 and 19p. Achkar and Duerr [29] identified IL23R and ATG16L1 as being involved in CD. These two genes are frequently mentioned in the existing literature [30]. Stoll et al. [31] identified DLG5, while Cleynen et al. [32] identified 163 susceptibility loci for IBD. Ahmad et al. mentioned that CD and UC are related diseases that share some but not all the susceptibility genes [33]. Inflammatory bowel disease is a chronic disease that typically requires lifelong medication [34]. Given the heterogeneity in the illness, it is not surprising that there are multiple treatment options with different levels of expected success.

Machine learning techniques are increasingly popular in medicine with applications in many different types of illness [35,36,37]. There has been some interesting research applying machine learning techniques in the context of inflammatory bowel disease [38,39,40]. This has been in part due to the large amount of data generated experimentally [41] and the need to come up with appropriate techniques to analyze such a large quantity of data. For instance, Wei et al. [42] used GWAS data to carry out a risk assessment of patients with ulcerative colitis or Crohn’s disease. Isakov et al. [43] identified 67 genes using machine learning techniques related to IBD. Coelho et al. [44] also used machine learning techniques, but their analysis covers pediatric patients, who have some characteristics different from the usual adult case. The same group of authors published another interesting paper [38] using three different machine learning techniques and endoscopic data, achieving an accuracy of 71.0%, 76.9% and 82.7% respectively. The work of Smolander et al. [45] is another interesting paper analyzing gene expression, using machine learning techniques in the context of complex disorders. Some authors, such as Stankvic et al. [46], mentioned that despite an increase in the use of machine learning techniques in IBD, the understanding of the illness remains incomplete.

One of the main objectives of this article is trying to identify genes that are relevant in the context of inflammatory bowel disease using machine learning techniques. The genes are chosen by selecting those genes with a gene expression level that is empirically useful to distinguish between control individuals and patients with IBD. The details of this process will be explained in the next section, but it is based on using different machine learning techniques (classification purposes) in combination with Monte Carlo simulations for the selection of genes. Another objective of this article is to be able to identity appropriate genes differentiating between Crohn’s disease and ulcerative colitis using a similar approach than when distinguishing between healthy and IBD patients.

## 2. Materials and Methods

**Figure 3:**
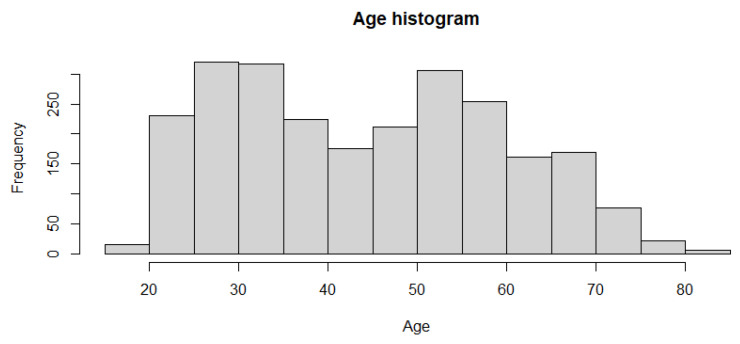
Histogram describing the age of the patients. The range is from 19 to 82 years old.

The dataset was retrieved from the Gene Expression Omnibus. The identification number is GSE 193677 [47]. The data include 2490 total cases. Of these 2490 cases, 461 are controls cases, while 2029 are individuals with adult inflammatory bowel disease (IBD). Of those 2029, a slight majority of 1157 have Crohn’s disease while 872 have ulcerative colitis. The average age of the patient is 44.9 years, with a range from 19 to 82 years old. A histogram showing the age distribution is shown in Figure 3. There are 1174 female and 1316 male cases. Tissue biopsies were obtained in the right colon, left colon, transverse, rectum, Ileum, sigmoid and cecum. The number of cases for each of this regions is summarized below in Table 1. The data consist of gene expression profiling by high throughput sequencing obtained using the Illumina HiSeq 2500. There are 56,632 expression profiling data per patient.

The data were divided into two subgroups, a training dataset and a testing dataset. $\Psi_{Tr}$ denotes the training dataset and $\Psi_{Ts}$ the testing dataset. The training and testing datasets contain approximately 80% and 20% of all the cases, respectively. Each column represents a patient. The division into a training and a testing dataset was carried out in a randomized way to try to avoid introducing biases in the analysis. The first row in each dataset contains a numerical classifier identifying the subject as a control or patient (UD or CD) as shown in Equation (1):

(1) $$\forall j\in \left[1,n\right],\;\Phi_{j}=\left\{Control=0,UC=CD=1\right\}$$

with *n* being the total number of cases. An example, for clarity purposes, can be seen in Equation (2):

(2) $$\Phi =\left\{\Phi_{1},\Phi_{2},\ldots ,\Phi_{n}\right\}=\left\{1,0,\ldots ,1\right\}$$

The following two rows contain the age (a), see Equations (3) and (4), and the gender (S), see Equations (5) and (6), of each individual, respectively:

(3) $$\forall j\in \left[1,n\right],a_{j}=\left\{x_{j}\right\}\;x\in R$$

(4) $$a=\left\{age\right\}=\left\{a_{1},a_{2},\ldots ,a_{n}\right\}=\left\{47,52,\ldots ,61\right\}$$

(5) $$\forall j\in \left[1,n\right],S_{j}=\left\{Female=0,Male=1\right\}$$

(6) $$S=\left\{gender\right\}=\left\{S_{1},S_{2},\ldots ,S_{m}\right\}=\left\{0,1,\ldots ,1\right\}$$

In a similar way, the following row contains the region for the biopsy. All the other rows contain gene expression data (see Equations (7) and (8)):

(7) $$\forall j\in \left[1,n\right],\forall k\in \left[1,m\right],g_{kj}=\left\{Z_{kj}\right\}\;Z\in R$$

(8) $$G_{k}=\left\{g_{kj}\right\}=\left\{18,241,\ldots ,132\right\}$$

where *k* is the index for each row. An example, for visualization purposes, of the data can be seen in Equation (9):

(9) $$\Psi_{Tr}=\left(\begin{array}{ccccc} \;0 & 1 & 2 & 0 & \cdots \\ 60 & 45 & 35 & 55 & \cdots \\ 0 & 0 & 1 & 1 & \cdots \\ 0 & 0 & 0 & 1 & \cdots \\ 80 & 30 & 55 & 40 & \cdots \\ \vdots & \vdots & \; & \vdots \\ \; \end{array}\right)$$

As a first step, the correlation $C_{0}\left(c,d\right)$ between the categorical data representing the classification group (control or IBD) and each row is calculated (Equations (10)):

(10) $$\forall \;k\in \left[1,m\right],C_{0}=C_{0}\left(\Phi ,G_{k}\right)$$

Therefore, $C_{0}$ is a vector with *m* components. From this mapping, the highest q%(0≤q≤100) is selected among these *m* values. Hence, there is a reduction in the dimension of the vector (Equation (11)):

(11) $$C_{0}\left(dim=m\right)\Rightarrow C_{0}^{*}\left(dim=m<k\right)$$

This step is performed in an attempt to include the factors that are potentially able to generate an accurate model while filtering out potential noise (not all genes are involved in inflammatory bowel disease). In other words, it is an attempt to filter out noise from genes than have no biological impact on the disease but that can lead the model to find spurious relationships given the large amount of data. The above-mentioned step is carried out only with the training dataset (containing approximately 80% of the cases). After this step, when the genes have already been selected, then all the other genes will be excluded from both the training and the testing dataset. In this way, it is possible to carry out a filtering of the initial gene list. A selection of 23 machine learning techniques was selected; see Table 2. Ten times cross validation was carried out (training dataset).

The artificial neural network (ANN) is a well-known machine learning algorithm. Given its versatility and wide use, this technique is used to determine a baseline classification accuracy, against which the other techniques are compared. In the ANN approach, it is necessary to carry out hyperparameter optimization. One of the key parameters to optimize is the number of layers in the ANN. This is achieved by carrying out simulations from 1 to 1000 layers and the related accuracy estimated. Unless explicitly mentioned, the accuracy (and other measures of the goodness of the fit) is that of the testing dataset (not used during the training phase). In this way, for each configuration γ(γ={1,…,1000}), an accuracy $A_{nn}$ measure is estimated $\left(A_{nn}^{\gamma }\right)$. Then, the best model $\left({\bar{A}}_{nn}\right)$ is selected as

(12) $${\bar{A}}_{nn}\left(\gamma \right)=sup\left(A_{c}^{\gamma }\right)$$

This is the baseline model. For each machine learning techniques, the model is trained with the training dataset, and then an accuracy estimate is obtained, and the best model $\bar{A}\left(\lambda \right)$ is selected (Equation (13)). The training and model selection (gene selection) is entirely performed with the training dataset. After the model is selected (including the genes), the accuracy and other metrics are expressed in terms of the testing dataset (not used for training or model selection):

(13) $$\bar{A}\left(\lambda \right)=sup\left(A^{\lambda }\right)$$

Then this is compared to the base level, selecting the final best model ${\bar{A}}_{max}$ as follows:

(14) $$A_{max}=max\left\{{\bar{A}}_{nn}\left(\gamma \right),\bar{A}\left(\lambda \right)\right\}$$

This analysis is initially carried out for all the gene expression data available after selecting the top q=1%. In this case, the initial number of gene expression data per patient entails 566 rows of information. Then a Monte Carlo approach is followed, in which the number of rows is randomly reduced in each iteration by a random number β. This random number β is changed in each iteration and is strictly less than the total number of rows in the previous iteration. An example is summarized in Table 3. The rationale behind using a Mote Carlo simulation approach is that it is not feasible to estimate all the possible combinations of 566 genes, and hence some type of combinatorial approach needs to be used. This is a frequent situation in polygenic illness, such as IBD, in which a potentially large number of genes might be involved in the disease.

This process is repeated *p* times (*p* = 100), and the ten most accurate models are selected.

In the second section, a similar approach is followed but the mapping shown in Equation (1) has to be changed, as the objective is now to distinguish between ulcerative colitis and Crohn’s disease cases (the two major types of IBD). The mapping in this case is as follows (Equation (15)):

(15) $$\forall j\in \left[1,u\right],\;\Phi_{j}=\left\{UC=0,CD=1\right\}$$

An alternative approach to the one presented is using a linear approach, such as, for instance, lasso regression [48,49]. Lasso regression offers the advantage that it makes some of the coefficients equal to zero, in practice reducing the number of inputs to the model. Using lasso regression, it is possible to reduce the number of genes selected for the classification model. In fact, lasso has become a frequently used feature selection algorithm [50,51].

## 3. Results

**Figure 4:**
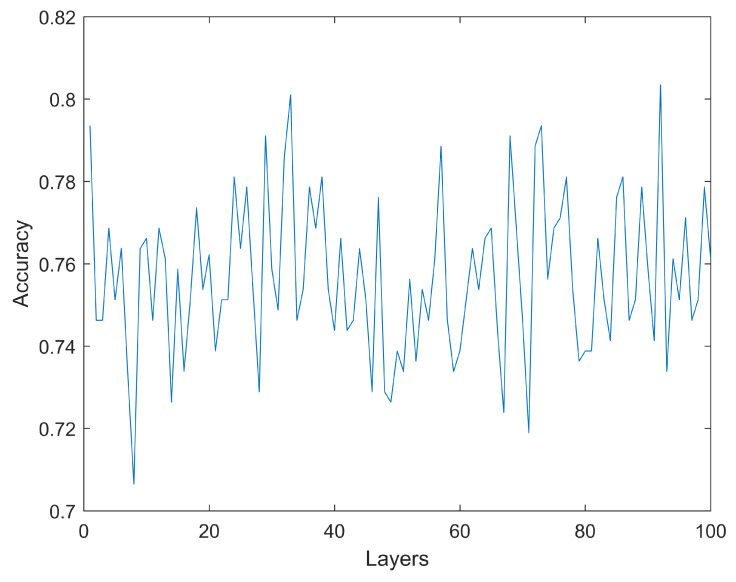
Accuracy of the neural network model for a range of number of artificial neurons. No model has an accuracy below 70% or higher than 80.35%.

As previously described, the first step involves estimating a base level for the accuracy using artificial neural networks with simulations using 1 to 100 hidden layers. Each layer consists of 30 neurons. As it can be seen in Figure 4, increasing the number of layers does not necessarily translate into higher accuracy. The highest accuracy (testing dataset) obtained is 80.35% with a configuration including 920 hidden layers. The only other simulation reaching an accuracy above 80.00% is an ANN with 330 layers, reaching 80.10%. All the other simulations achieve a mean accuracy below 80.00%. No model has an accuracy below 70%. These results are obtained for a configuration of 74 rows (gene expression) which, as will be shown later, is the configuration that obtains the highest accuracy for the machine learning algorithm tested. As previously mentioned, the reported accuracy is the accuracy of the testing dataset, which is not used during the training phase.

Different machine learning algorithms are tested (as described in the Materials and Methods section). As an example, in Table 4, the accuracy results for one of the simulations are shown (140 gene expressions). In this specific case, the highest accuracy obtained is 81.5%. This accuracy is obtained by five different algorithms (Linear SVM, Fine Gaussian SVM, Medium Gaussian SVM, Coarse Gaussian SVM and Coarse KNN).

The results from the 10 most accurate simulations can be seen in Table 5. Of the ten most accurate results, nine use the bagged trees algorithm. The only other algorithm in the top ten most accurate models is the Subspace KNN. The highest accuracy is obtained for a model with 74 gene expression data, obtaining an accuracy, sensitivity and specificity of 84.2%, 82.6% and 84.4%, respectively. The list with these 74 genes can be found in Table 6.

The results, when differentiating UC and CD cases, are not as accurate as when differentiating between control cases and IBD cases. This is in line with the expectations, as we are differentiating between two types of the same illness. These results are shown in Table 7. The most accurate result is obtained when using 562 gene expression data and the bagged trees algorithm. The accuracy, sensitivity and specificity are 73.4%, 79.0% and 71.2%, respectively. The list with these 562 genes can be found in the Supplementary Material.

As previously mentioned, an alternative approach to the one proposed is using lasso regression as a tool for the selection of inputs. The lasso approach selects 470 genes with the goodness-of-fit metric shown in Table 8. The accuracy and specificity results obtained in this approach are similar to those obtained in the proposed approach in the previous section. However, the sensitivity results from the lasso approach seem to be lower.

The lasso approach is also used to distinguish between UC and CD patients. In this case, the lasso approach selects 430 genes. The table with the goodness-of-fit results in this approach is shown below (Table 9). The results using the lasso approach to distinguish between UC and DC patients are not as accurate as in the previous section. In both cases, using lasso or the proposed approach, differentiating between UC and DC patients appears to be more challenging than differentiating between control health individuals and patients with UC/CD. The lasso approach does not appear to increase the goodness of fit of the classification forecasts compared to the approached followed in the previous section.

## 4. Discussion

Machine learning techniques are used to identify a set of 74 genes, which can be used, with an average accuracy of 84.2%, to distinguish between control (healthy individuals) and patients with inflammatory bowel disease. The specificity and sensitivity of this model are also relatively high at 82.6% and 84.4%, respectively. The selection of these 74 genes is carried out following a Monte Carlo simulation approach. Given that some of the symptoms of inflammatory bowel disease are common in other illnesses, it might be interesting to have another objective diagnostic tool. It is also interesting to observe that among multiple machine learning techniques used in the cohort of patients analyzed, the bagged trees approach seems to consistently achieve a high level of accuracy, particularly when compared to other, arguably more sophisticated machine learning techniques, such as artificial neural networks. The analysis controls for age, gender and region of the biopsy. The proportion of female and male cases is balanced, with 1174 female patients and 1316 male patients. The average age in the cohort is 44.9 years, covering a wide age range (from 19 to 82 years old). The results of the artificial neural networks include an optimization of the hyperparameters with simulations ranging from 1 to 1000 hidden layers. It is also observed that simply increasing the number of layers in an artificial neural network does not necessarily translate into better accuracy. It is also possible to distinguish between the two main types of IBD—Crohn’s disease and ulcerative colitis—but in this case with a lower level of accuracy. The accuracy, using this approach is 73.4%. The accuracy, sensitivity and specificity reported are those of the testing dataset. As normal practice, the data are divided into training and testing datasets in an attempt to increase the reproducibility of the analysis. Approximately 20% of the total cases are included in the testing dataset. The relatively large number of genes obtained in the bets model is in line with the prevalent view in the existing literature that the illness is polygenic.

There is a high degree of heterogeneity in inflammatory bowel disease, leading to varied severity and evolution of the illness. The existing literature, see, for instance, Yamamot et al. [52] or Ahmad et al. [33], points towards a polygenic illness with a complex interaction with environmental factors. Our results are consistent with this polygenic description. In this context, it is important to generate algorithms that are able to differentiate among control and patients as well as between different types of inflammatory bowel disease, namely Crohn’s disease and ulcerative colitis. A promising area of future research is to apply this type of approach in order to target treatments in a more personalized way. It seems reasonable that there could be genetic differences among patients that can have a substantial impact on the outcome of the suggested treatments. This is particularly important in the context of inflammatory bowel disease, given the heterogeneity of the responses to treatments by different patients.

Some of the genes identified by the proposed algorithm are cited in the existing literature on intestinal-related illnesses. B2M was mentioned by Krzystek-Korpacka et al. [53] in the context of bowel inflammation. There are other papers, such as that of Bednarz-Misa et al. [54], discussing B2M in the context of bowel inflammation and cancer. Another gene identified by the algorithm is MALAT1, which is also mentioned in the existing literature. Li et al. [55] suggested that MALAT1 maintains intestinal mucosal homeostasis in Crohn’s disease. The authors concluded that the downregulation of MALAT1 contributes to the pathogenesis of CD. EEF1A1 was identified in a dog study as being involved in inflammatory bowel disease and cancer by Sahoo et al. [56]. The role of MUC2 in protecting the integrity of the mucosa was mentioned by Huang et al. [57]. The authors mentioned that it is possible to induce colitis in mice by suppressing the MUC2 gene. Heimel et al. [58] found high levels of expression of FABP2 and FABP6 when analyzing alterations in intestinal fatty acid metabolism in IBD. CA1 was mentioned by Xie et al. [59] as playing a role in IBD. PHGR1 was identified by Camilleri et al. [60] as potentially increasing the risk of diverticular disease of the colon. FABP1 was identified as a biomarker for Crohn’s disease by Dooley et al. [61]. COL1A2 was mentioned by Prados et al. [62] in murine models of IBD. ENO1 was mentioned by Shkoda et al. [63] for its role in IBD pathobiology. Another gene selected by the algorithm and mentioned in the literature as being related to IBD is NDRG1 [64]. Song et al. [65] showed that ADH1C is downregulated in UC. FN1 was suggested by Al-Numan [66] to be related to the early onset of IBD. SPINT2 plays a role in epithelial adhesion [17]. CLDN7 is associated with colitis according to several authors [67,68]. Darsigny et al. [69] found a link between APOC3 and chronic inflammation in mice resembling IBD. KLF5 was identified by Dong et al. [70] as one of the genes downregulated in IBD. Gorenjak et al. [71] linked HSPA9 with IBD.

One of the challenges, and possible limitations, of this type of analysis is the fact that it is impossible to estimate all possible combinations of genes, and hence it is necessary to use some sort of combinatorial approach, such as the Monte Carlo model used to select the genes. There is also no indication that gene expression and IBD are related by an underlying linear model. Given this assumption, using machine learning techniques, which are adept to modeling nonlinear systems, seems like a reasonable approach. Another factor to take into account is that, while the cohort of cases is not small, including 2490 cases, it can always be larger.

## 5. Conclusions

Following a machine learning approach, it was possible to identify a list of genes that appear to be related to inflammatory bowel disease. Given the complexity of this illness, which appears to be caused by a combination of polygenic factors as well as environmental factors, which could, in principle, interact in a non-linear way, the illness was analyzed using non-linear models, such as machine learning techniques. This approach was able to distinguish, using a small number of genes, between patients with IBD and control (healthy) patients as well as patients with the two major forms of IBD, which are Crohn’s disease and ulcerative colitis. In other words, the machine learning algorithms are able to classify different types of gene expression signatures associated with IBD. It might be possible in the future, when more data become available, to be able to distinguish between different genetic signatures of the illness that might potentially help develop more personalized treatments. This is important for an illness as heterogeneous as IBD, for which patients follow different evolutions and might present different clinical manifestations.

## Figures and Tables

**Table 1:** Biopsies (tissue areas).

Area	Cases
Rectum	904
Left colon	180
Right colon	252
Ileum	672
Transverse	90
Sigmoid	163
Cecum	229

**Table 2:** Selected machine learning algorithms.

Algorithm	Algorithm
Complex Tree	Fine KNN
Medium Tree	Medium KNN
Simple Tree	Coarse KNN
Linear Discriminant	Cosine KNN
Quadratic Discriminant	Cubic KNN
Logistic Regression	Weighted KNN
Linear SVM	Boosted Trees
Quadratic SVM	Bagged Trees
Cubic SVM	Subspace Discriminant
Fine Gaussian SVM	Subsspace KNN
Medium Gaussian SVM	RUSBossted Trees
Coarse Gaussian SVM	

**Table 3:** Example of iterative algorithm testing different configurations of gene expressions.

Iteration	Initial N. Genes	β
0	566	30
1	536	125
2	411	58
3	353	9
4	344	215
⋮	⋮	⋮

**Table 4:** As an example, in this table, sample training with all 23 algorithms is shown. In this case, the model uses 140 gene expression data and the highest accuracy is 81.5%. This accuracy is actually reached by several algorithms (Linear SVM, Fine Gaussian SVM, Medium Gaussian SVM, Coarse Gaussian SVM and Coarse KNN).

Algorithm	Accuracy
Complex Tree	0.701
Medium Tree	0.783
Simple Tree	0.804
Linear Discriminant	0.645
Quadratic Discriminant	0.711
Logistic Regression	0.807
Linear SVM	0.815
Quadratic SVM	0.788
Cubic SVM	0.756
Fine Gaussian SVM	0.815
Medium Gaussian SVM	0.815
Coarse Gaussian SVM	0.815
Fine KNN	0.719
Medium KNN	0.770
Coarse KNN	0.815
Cosine KNN	0.768
Cubic KNN	0.764
Weighted KNN	0.773
Boosted Trees	0.805
Bagged Trees	0.804
Subspace Discriminant	0.812
Subsspace KNN	0.748
RUSBossted Trees	0.606

**Table 5:** Top ten models obtained according to the accuracy metric.

N. Genes	Algorithm	Accuracy	Sensitivity	Specificity
74	Bagged Trees	0.842	0.826	0.844
38	Subspace KNN	0.842	0.755	0.859
18	Bagged Trees	0.839	0.787	0.847
139	Bagged Trees	0.836	0.755	0.850
220	Bagged Trees	0.834	0.758	0.847
266	Bagged Trees	0.833	0.740	0.850
26	Bagged Trees	0.833	0.821	0.834
16	Bagged Trees	0.833	0.879	0.828
17	Bagged Trees	0.831	0.738	0.848
104	Bagged Trees	0.830	0.750	0.843

**Table 6:** List of 74 genes selected by the algorithm.

B2M	RPS3	CHP1	SLC35A3
MALAT1	MAN2B1	ETNK1	PDIA3
EEF1A1	NDRG1	SLC1A2	DDX3X
MUC2	AHCYL2	GHITM	WDR1
FABP6	RPS14	MGAT4A	KLF5
KRT20	MYO1D	CLDN7	TSC22D1
CA1	A2M	COPZ2	RPL35A
FLNB	ADH1C	APOC3	SCP2
PHGR1	DDX17	SAT1	MATR3
IGKV1-5	FOS	ACE	CD46
CKB	RPL7	CD2AP	HNRNPH1
FABP1	SLC44A1	PAPSS2	PRKDC
FABP2	FN1	PDCD4	RPL37
CLDN4	RPL18	HPGD	LUM
TSPAN3	TDP2	UGT2A3	HSPA9
CDHR2	RPS12	UQCRC1	KIAA1109
CLTC	SPINT2	ST6GALNAC6	MIM24
COL1A2	RPL10A	ARF1	
ENO1	NCOA4	PRKACB	

**Table 7:** Top ten models obtained according to the accuracy metric distinguishing UC and CD patients.

N. Genes	Algorithm	Accuracy	Sensitivity	Specificity
562	Bagged Trees	0.734	0.790	0.712
66	Bagged Trees	0.728	0.679	0.767
24	Bagged Trees	0.718	0.665	0.742
37	Bagged Trees	0.718	0.821	0.687
564	Bagged Trees	0.712	0.909	0.671
132	Bagged Trees	0.704	0.929	0.676
49	Bagged Trees	0.704	0.679	0.719
15	Bagged Trees	0.700	0.713	0.697
550	Bagged Trees	0.694	0.871	0.659
277	Bagged Trees	0.673	0.616	0.717

**Table 8:** Top ten models obtained using the lasso approach (470 genes) according to the accuracy metric distinguishing between control and UC and CD patients.

Algorithm	Accuracy	Sensitivity	Specificity
Medium KNN	0.817	0.667	0.817
Bagged Trees	0.817	0.667	0.817
Weighted KNN	0.815	0.500	0.816
Cubic KNN	0.807	0.143	0.815
Simple Tree	0.804	0.231	0.816
Subspace Dis.	0.804	0.405	0.829
Linear Dis.	0.802	0.433	0.842
Cosine KNN	0.802	0.300	0.819
Medium Tree	0.797	0.200	0.817
Subspace KNN	0.786	0.313	0.826

**Table 9:** Top ten models obtained using the lasso approach (430 genes) according to the accuracy metric distinguishing between UC and CD patients.

Algorithm	Accuracy	Sensitivity	Specificity
Subspace Dis.	0.584	0.611	0.523
Logistic Reg.	0.572	0.617	0.503
Medium KNN	0.568	0.580	0.493
Cubic KNN	0.562	0.578	0.474
Weighted KNN	0.560	0.584	0.478
Simple Tree	0.558	0.569	0.412
Bagged Trees	0.558	0.583	0.474
Boosted Trees	0.556	0.580	0.467
Cosine KNN	0.550	0.574	0.448
Fine KNN	0.538	0.595	0.463

## Data Availability

The data are accessible at: https://www.ncbi.nlm.nih.gov/geo/ (accessed on 1 June 2023).

## Supplementary Materials

The following supporting information can be downloaded at: https://www.mdpi.com/article/10.3390/medicina59071218/s1, Supplementary Material: The list of 562 genes.

## Abbreviations

The following abbreviations are used in this manuscript:

IBD	Inflammatory Bowel Disease (IBD)
UC	Ulcerative Colitis
CD	Crohn’s Disease
GEO	Gene Expression Omnibus
KNN	k-Nearest Neighbors
SVM	Support Vector Machine
ANN	Artificial Neural Network
